# Role of Esterase BmeFE4 in Mediating the Alleviating Effects of Resveratrol on NaF-Induced Fluorosis in *Bombyx mori*

**DOI:** 10.3390/toxics14090829

**Published:** 2026-09-18

**Authors:** Liang Chen, Qilong Zhao, Mengyue Wang, Tongyu Gu, Lei Ding, Xinzhi Wang, Qi Tang, Shangshang Ma, Guorui Liu, Keping Chen

**Affiliations:** 1 School of Biological Science and Technology, Jiangsu University, Zhenjiang 212013, China; 2212417032@stmail.ujs.edu.cn (Q.Z.); 3234501025@stmail.ujs.edu.cn (M.W.); 18456074978@163.com (T.G.); 2212117012@stmail.ujs.edu.cn (L.D.); tangqi1224@163.com (Q.T.); mashang@ujs.edu.cn (S.M.); 3234501001@ujs.edu.cn (G.L.); 2 State Key Laboratory of Natural Medicines, New Drug Screening Center, China Pharmaceutical University, Nanjing 210009, China; wangxz@cpu.edu.cn

**Keywords:** fluorosis, resveratrol, transcriptome, proteome, association analysis

## Abstract

Fluoride exposure can induce tissue injury and oxidative stress dysregulation, ultimately leading to organismal dysfunction and death, whereas resveratrol (RSV) has been reported to counteract diverse oxidative injuries. Despite its antioxidant potential, the application of RSV is limited by its low bioavailability. Here, using a silkworm model, we established NaF exposure, RSV treatment, and combined RSV+NaF groups to investigate the mechanism by which RSV attenuates fluorosis. Comparative analyses of body weight, histopathological damage, and oxidative stress indices showed that RSV markedly mitigated NaF-induced toxicity. Candidate genes and proteins exhibiting concordant expression changes were identified through integrated RNA-seq and TMT-based quantitative proteomics, and subsequently validated by qPCR and PRM. Among these candidates, the esterase BmeFE4 emerged as a key regulator of the RSV-mediated detoxification response. Spatiotemporal profiling revealed that *BmeFE4* was expressed throughout silkworm development, with the highest levels in the midgut. RNAi-mediated knockdown of *BmeFE4* significantly altered NaF-related phenotypes and modulated oxidative-stress-related parameters, including superoxide dismutase (SOD) and catalase (CAT) activities, reduced glutathione (GSH) content, and malondialdehyde (MDA) levels, as quantified by ELISA. Collectively, these results indicate that RSV alleviates fluoride toxicity at least in part by upregulating *BmeFE4*, identifying this esterase as a potential molecular target for developing defluoridation agents.

## 1. Introduction

Fluoride is a widespread environmental pollutant of concern. Excessive fluoride exposure can cause irreversible damage to tissue structure and disrupt metabolism in plants, animals, and humans, resulting in substantial economic losses [1,2,3,4]. From an ecological and toxicological perspective, fluoride overexposure is cardiotoxic in mammals and can impair reproductive function [5,6]. Fluoride is absorbed mainly through the gastrointestinal tract. At appropriate levels, fluoride contributes to enzymatic metabolism and neural conduction and promotes mineralization of teeth and bone. However, chronic daily intake above ~6 mg in humans has been associated with an increased risk of fluorosis [7,8]. Accordingly, considerable efforts have been devoted to developing remediation strategies. At the environmental level, fluoride-accumulating plants have been cultivated to remove fluoride from contaminated settings [9]. From a diet therapy perspective, certain plant-derived natural products such as resveratrol (RSV) [10,11], quercetin [12,13], and plant polyphenols [14,15] have been found to alleviate fluorosis symptoms. These natural products can effectively mitigate fluorosis by reducing oxidative stress, utilizing detoxifying enzymes, and influencing signaling pathways [16,17,18,19]. A key remaining challenge is to identify the specific molecular targets that mediate detoxification responses in exposed organisms.

The emergence of multi-omics analysis has coincided with the widespread adoption of high-throughput technology [20,21]. This enables researchers to collect extensive molecular data across multiple molecular levels, such as the genome, transcriptome, proteome, epigenome, metabolome, and microbiome [22,23,24,25]. RNA sequencing (RNA-seq) enables genome-wide profiling of transcript abundance, whereas tandem mass tag (TMT)-based quantitative proteomics allows multiplexed comparisons of relative protein abundance across experimental groups. The integration and analysis of multi-omics data facilitate a comprehensive comprehension understanding of biological processes and molecular mechanisms [26,27,28]. By comparing molecular changes at the transcript and protein levels, this strategy can increase confidence in candidate selection and narrow the range of targets for subsequent validation and functional analysis. Consequently, the emergence of multi-omics technologies accelerates more efficient drug target discovery and the identification of more effective biomarkers.

*Bombyx mori* is easy to raise, has a short life cycle, and possesses clear genomic information, making it a highly promising model organism. In our previous study, we discovered that exposing the larvae of *Bombyx mori*, commonly known as silkworms, to a solution of sodium fluoride (NaF) at a concentration of 600 mg L^−1^ resulted in significant fluorosis [29]. This phenomenon was thoroughly investigated using multi-omics techniques to explore the potential target genes affected by fluoride in *Bombyx mori*. Moreover, our previous studies have also revealed that 5 mM of RSV could effectively extend the lifespan of silkworms.

In this study, we examined the effects of RSV administered before and during NaF exposure in *Bombyx mori*. Specifically, we investigated the protective effects of RSV against fluorosis induced by NaF and the underlying mechanisms involved. Through RNA-seq and Tandem Mass Tag (TMT) techniques, we identified that BmeFE4 plays a pivotal role in protecting against NaF-induced fluorosis and serves as a significant molecular target for mitigating fluorosis with RSV. Our findings will contribute to a better understanding of the molecular mechanisms associated with NaF-induced fluorosis and offer novel insights into protective strategies against fluoride toxicity.

## 2. Materials and Methods

### 2.1. Silkworm Rearing, Modeling, and Treatment

Sodium fluoride was purchased from Shenggong (Shanghai, China). Resveratrol was procured from Sigma (St. Louis, MO, USA). The experimental domestic silkworm strain was supplied and maintained by the School of Life Sciences, Jiangsu University. Double-deionized water was used throughout the experiment. Resveratrol (RSV) was prepared by dissolving 0.23 g RSV in 10 mL dimethyl sulfoxide (DMSO) to obtain a 100 mM stock solution, which was subsequently diluted to 5 mM as the working solution, resulting in a final DMSO concentration of 5% (*v*/*v*). A sodium fluoride (NaF) treatment solution was prepared by dissolving 0.60 g NaF in 1 L distilled water to a final concentration of 600 mg·L^−1^.

Silkworm larvae were reared under standard conditions (25 °C, 65–75% relative humidity, 12 h light/12 h dark photoperiod) and fed mulberry leaves. For experimental treatments, fresh mulberry leaves were collected from a mulberry garden, rinsed with distilled water, and air-dried before use [30]. After reaching the 4th instar, larvae were randomly assigned to four groups: (i) control, fed untreated mulberry leaves; (ii) NaF, fed mulberry leaves treated with 600 mg·L^−1^ NaF solution starting from the 5th instar; (iii) RSV, continuously fed mulberry leaves treated with 5 mM RSV starting from the 4th instar; and (iv) RSV+NaF, fed mulberry leaves co-treated with 600 mg·L^−1^ NaF and 5 mM RSV during the 5th instar. On day 6 of the 5th instar, larvae from all groups were dissected, midgut tissues were collected, immediately snap-frozen in liquid nitrogen, and stored in liquid nitrogen until further analyses.

### 2.2. Determination of Physiological and Biochemical Indices and Observation of Tissue Sections

The body weight and morphology of the larvae in each group were photographed and recorded daily starting from the fifth instar. On the sixth day of the fifth instar, random samples were taken from each group for the determination of oxidative-stress-related parameters, hematoxylin and eosin (H&E) staining, and histological analysis, following the method described by Chen et al. [29]. The dissected midgut tissues were promptly washed twice in a pre-cooled phosphate-buffered saline (PBS) solution at 4 °C. Excess liquid was removed from the tissues using filter paper. The tissues were then placed into 1.5 mL Eppendorf (EP) tubes, quickly frozen in liquid nitrogen, and preserved in a −80 °C refrigerator.

### 2.3. RNA-Seq and TMT Sequencing Analysis and Validation

Midgut samples from the control, NaF-exposed, RSV-treated, and NaF+RSV co-treated groups were subjected to RNA sequencing and TMT protein quantification for screening and functional annotation of differentially expressed genes (DEGs) and differentially expressed proteins (DEPs). For RNA-seq, the HiSeq 2000 sequencing platform (Illumina, Inc., San Diego, CA, USA) was used to obtain raw reads. These raw reads underwent quality control using SeqPrep v1.2 and Sickle v1.33 software to obtain high-quality clean reads. Afterward, the NOIseq v2.18.0 software was utilized to screen for DEGs between the groups. TMT protein quantification involved collecting proteins through liquid chromatography and mass spectrometry (LC-MS/MS) and then utilizing the Proteome Discoverer v2.2 (Thermo Fisher Scientific, Waltham, MA, USA) module to search the library for protein identification.

### 2.4. Collaborative Transcriptome and Proteome Analysis and Validation

The Control_vs_NaF gene set, NaF_vs_DZ protein set, NaF_vs_RSV_NaF gene set, and RSV_NaF_vs_NaF protein set were subjected to Venn analysis, respectively. Before the integrated analysis, DEGs from the RNA-seq dataset were screened using the criteria |log2FC| ≥ 1 and prob < 0.8, whereas DEPs from the TMT-based proteomic dataset were identified using *p* < 0.05 and (FC > 1.1 or FC < 0.83). Genes and proteins present in the corresponding intersections and exhibiting concordant expression trends were selected for validation; primer sequences are provided in Table S1.

### 2.5. Spatiotemporal Expression of BmeFE4

Based on our pre-transcriptome data for the silkworm (BMIBGMA002899) (https://www.ncbi.nlm.nih.gov/sra/PRJNA838458, accessed on 17 September 2026), gene sequences were obtained from the Open Reading Frame Finder website (https://www.ncbi.nlm.nih.gov/orffinder, accessed on 17 September 2026) and translated. The ExPAsy online website (https://www.expasy.org, accessed on 17 September 2026) was used to predict the isoelectric point and molecular weight of the amino acid sequences. Additionally, signal peptides were predicted using the SignalP-5.0 online website (https://services.healthtech.dtu.dk, accessed on 17 September 2026).

#### 2.5.1. Multiple Sequence Comparison and Phylogenetic Tree

The amino acid sequence of BmeFE4 was compared with carboxylesterases from different species using the blast of National Center for Biotechnology Information (NCBI) (https://blast.ncbi.nlm.nih.gov/Blast.cgi, accessed on 17 September 2026). Phylogenetic trees based on carboxylesterase sequences from other species were constructed using the neighbor-joining method in MEGA (v11.0) software.

#### 2.5.2. Expression of *BmeFE4* at Different Developmental Stages and in Different Tissues

Several normally growing and well-developing first to fifth-instar day 2 larvae, pupae, and moths were collected. They were rapidly frozen in liquid nitrogen and stored at −80 °C. Subsequently, RNA was extracted using TRIzol reagent (Invitrogen, Carlsbad, CA, USA), followed by reverse transcription and real-time quantitative polymerase chain reaction (RT-qPCR). RT-qPCR primer sequences for *BmeFE4* (BGIBMGA002899) are shown in Table S1.

Several dissected midguts, fat bodies, heads, Malpighian tubules, and filament glands from fifth instar day 3 larvae were washed twice in pre-cooled PBS solution, with any remaining liquid blotted off using absorbent paper. After freezing in liquid nitrogen, they were stored at −80 °C. RNA extraction was performed using the TRIzol method, followed by reverse transcription and RT-qPCR.

#### 2.5.3. Validation of Small Interfering RNA (siRNA)-Mediated Interference of *BmeFE4* in *Bombyx mori*

Based on the sequence of the *BmeFE4* gene, double-stranded RNA was designed and synthesized by Shanghai Bioengineering. Three segments of siRNA primers (siRNA-1, siRNA-2, siRNA-3) were designed in the pre-experiment, and the sequences of the interfering primers are shown in Table S2. From the results of the interference in the pre-experiment, the successful segment siRNA-3 was selected for subsequent experiments. The siRNA was solubilized by adding 70 μL of diethyl pyrocarbonate (DEPC) water per 1 optical density (OD). The larvae were divided into three treatment groups: (i) Experimental group: Larvae were fed normally until the 4th instar of silkworms. Afterward, the larvae were fed mulberry leaves sprayed with 5 mM RSV. Then, the 5th instar silkworm larvae with good growth conditions were selected. Using a microsyringe (Hamilton, Reno, NV, USA), 10 μL of siRNA was injected, and the injection site was at the penultimate joint of the larvae’s abdomen. Following the injection, the mulberry leaves were soaked in a 600 mg L^−1^ NaF solution for 15 min. Finally, the larvae were sprayed with 5 mM RSV and fed naturally; (ii) Negative control: The negative control primer sequences were provided by Shanghai Shenggong. The injection and feeding methods were the same as those of the experimental group; (iii) Control check: This group consisted of normal growth larvae injected with an equal amount of DEPC water.

After 12 h, 24 h, and 48 h of injection interference, the larvae were taken and stored in liquid nitrogen at −80 °C. Each group underwent three rounds of biological replication, with 15 silkworms injected per round.

#### 2.5.4. Enzyme Activity Assay of BmeFE4

The enzyme activity was determined using the enzyme-linked immunosorbent assay (ELISA), following the instructions provided with the enzyme activity assay kit (Shanghai Jining Biotechnology Co., Ltd., Shanghai, China).

### 2.6. Statistical Analysis

All experiments were repeated three times. The results were presented as the mean ± standard deviation (SD). GraphPad Prism 7.01 (GraphPad Software, La Jolla, CA, USA) and SPSS v24.0 (IBM Corp., Armonk, NY, USA) were used for statistical analysis. For body-weight data, differences among treatment groups at each sampling time point were analyzed separately using one-way analysis of variance (ANOVA), followed by Fisher’s least significant difference (LSD) post hoc test. A significance level of *p* < 0.05 was considered statistically significant. In figures using lowercase letters, groups sharing no letter differ significantly (*p* < 0.05).

## 3. Results

### 3.1. RSV Alleviated NaF-Induced Toxicity Phenotypes

**Figure 1:**
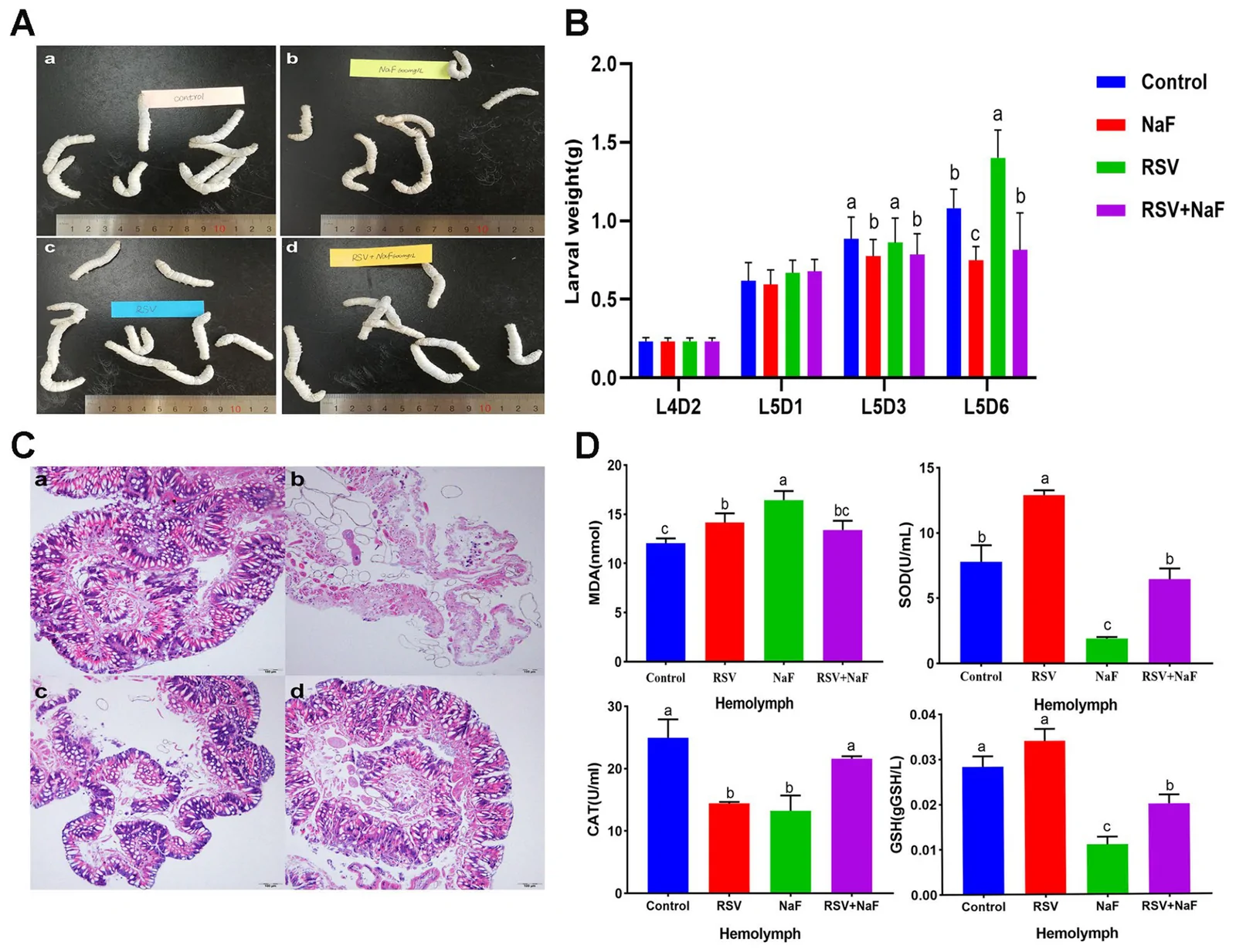
Effects of NaF exposure and resveratrol (RSV) treatment on silkworm phenotype, body weight, midgut histopathology, and oxidative-stress-related parameters. (**A**) Representative images of silkworms from the Control (**a**), NaF (**b**), RSV (**c**), and RSV+NaF (**d**) groups. (**B**) Body weight of silkworms under the indicated treatments. (**C**) Representative hematoxylin–eosin (H&E) staining of midgut sections. (**D**) Oxidative-stress-related parameters, including the MDA level, SOD and CAT activities, and GSH content. Group differences were analyzed by one-way ANOVA followed by Fisher’s least significant difference (LSD) post hoc test; *p* < 0.05 was considered statistically significant. Bars without a common lowercase letter differ significantly (*p* < 0.05).

As shown in Figure 1A,B, silkworms in the NaF group displayed pronounced abnormalities compared with the control group. They were markedly smaller, exhibited erratic crawling, consumed substantially less food, produced less frass, and showed a downward trend in body weight; however, the difference in body weight between the NaF and control groups did not reach statistical significance (*p* > 0.05). By contrast, RSV co-treatment (RSV+NaF) partially restored body weight toward control levels. Moreover, RSV alone increased body weight at L5D3 and L5D6 (*p* < 0.05 and *p* < 0.001, respectively). To assess whether RSV mitigated NaF-induced intestinal injury a qualitative histopathological assessment of midgut tissues was performed. As shown in Figure 1C, midguts from control silkworms exhibited densely packed microvilli, with columnar and goblet-like cells orderly arranged along the basement membrane. In the treated groups, NaF exposure was associated with reduced microvilli, cellular lysis, and cell death. Notably, qualitative examination of the representative H&E-stained sections showed that these histopathological alterations were less pronounced in the RSV+NaF group than in the NaF group, supporting a protective effect of RSV against NaF-induced intestinal injury.

Oxidative-stress-related parameters provide an important readout of organismal injury and help elucidate the biochemical effects of NaF exposure [31,32]. As shown in Figure 1D, malondialdehyde (MDA) levels were significantly elevated in the NaF group (16.438 ± 0.527), representing an ~1.36-fold increase relative to the control group (12.056 ± 0.283; *p* < 0.05). In the RSV+NaF group, MDA was lower (13.414 ± 0.538), corresponding to only an ~1.11-fold increase versus controls and an ~0.82-fold decrease compared with the NaF level. Conversely, superoxide dismutase (SOD) and catalase (CAT) activities and reduced glutathione (GSH) content were reduced in the NaF group (1.912 ± 0.064, 13.234 ± 1.424, and 0.011 ± 0.001, respectively) compared with the control group (7.784 ± 0.737, 24.962 ± 1.705, and 0.028 ± 0.001), corresponding to ~0.25-, ~0.53-, and ~0.39-fold of control levels. Functionally, SOD catalyzes the dismutation of superoxide radicals, CAT decomposes hydrogen peroxide, and GSH acts as a major non-enzymatic antioxidant that helps maintain cellular redox homeostasis. Notably, RSV co-treatment increased SOD, CAT, and GSH to 0.83-, 0.87-, and 0.71-fold of the respective control values (6.454 ± 0.475, 21.605 ± 0.225, and 0.020 ± 0.001), and to 3.38-, 1.63-, and 1.82-fold relative to the NaF group. Taken together, NaF exposure increased MDA levels and reduced SOD and CAT activities and GSH content, whereas RSV supplementation substantially reversed these changes, indicating that RSV alleviates NaF-induced oxidative stress in silkworms.

### 3.2. Screening, Functional Annotation, and qPCR Validation of Significant Differential Genes

To elucidate the mechanisms by which resveratrol (RSV) alleviates NaF-induced fluorosis in *Bombyx mori*, we performed integrated transcriptomic and proteomic analyses of midgut tissues from four experimental groups (*n* = 3 biological replicates per group; 12 samples total) collected on day 6 after NaF exposure to identify differentially expressed genes (DEGs) and proteins (DEPs).

Transcriptome sequencing generated 85.82 Gb of high-quality clean data. Each sample yielded >6.34 Gb, with >95% of bases at Q30, indicating robust sequencing quality (Table S3). Two comparison sets were constructed: Control vs. NaF and NaF vs. RSV+NaF. A total of 1493 DEGs were identified in the Control vs. NaF comparison, including 574 upregulated and 919 downregulated genes. In the NaF vs. RSV+NaF comparison, 1091 DEGs were identified, including 767 upregulated and 324 downregulated genes (Table S4). Venn analysis is shown in Figure S1. The overlap between genes upregulated in Control vs. NaF and downregulated in NaF vs. RSV+NaF comprised 178 genes (defined as the “G1” set). Conversely, the overlap between genes downregulated in Control vs. NaF and upregulated in NaF vs. RSV+NaF comprised 439 genes (defined as the “G2” set). The G1 and G2 sets were subsequently subjected to Gene Ontology (GO) and Kyoto Encyclopedia of Genes and Genomes (KEGG) enrichment analyses, respectively.

**Figure 2:**
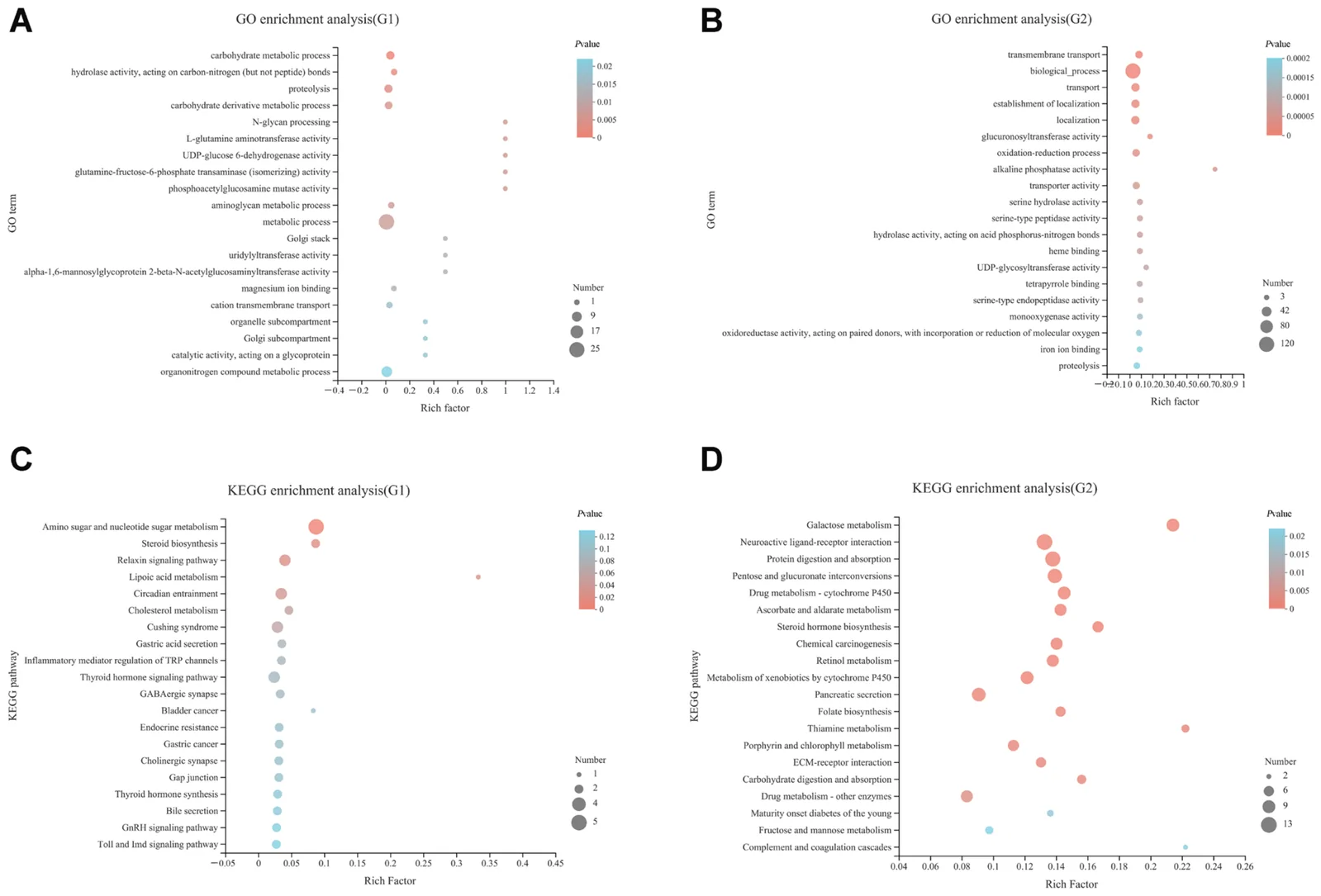
Functional enrichment of the G1/G2 gene set. (**A**) GO enrichment for G1 gene set; (**B**) GO enrichment for G2 gene set; (**C**) KEGG enrichment for G1 gene set; (**D**) KEGG enrichment for G2 gene set.

GO enrichment analysis indicated that the G1 gene set was predominantly associated with metabolic processes, including proteolysis/protein hydrolysis, carbohydrate-derivative metabolism, and carbohydrate metabolic processes (Figure 2A). In contrast, the G2 gene set was mainly enriched in biological processes related to enzymatic functions, including alkaline phosphatase activity, glucuronosyltransferase activity, and serine hydrolase activity (Figure 2B). Consistent with these findings, KEGG pathway analysis showed that G1 genes were primarily enriched in amino sugar and nucleotide sugar metabolism (Figure 2C), whereas G2 genes were mainly mapped to galactose metabolism, protein digestion and absorption, drug metabolism—cytochrome P450—and metabolism of xenobiotics by cytochrome P450 (Figure 2D).

**Figure 3:**
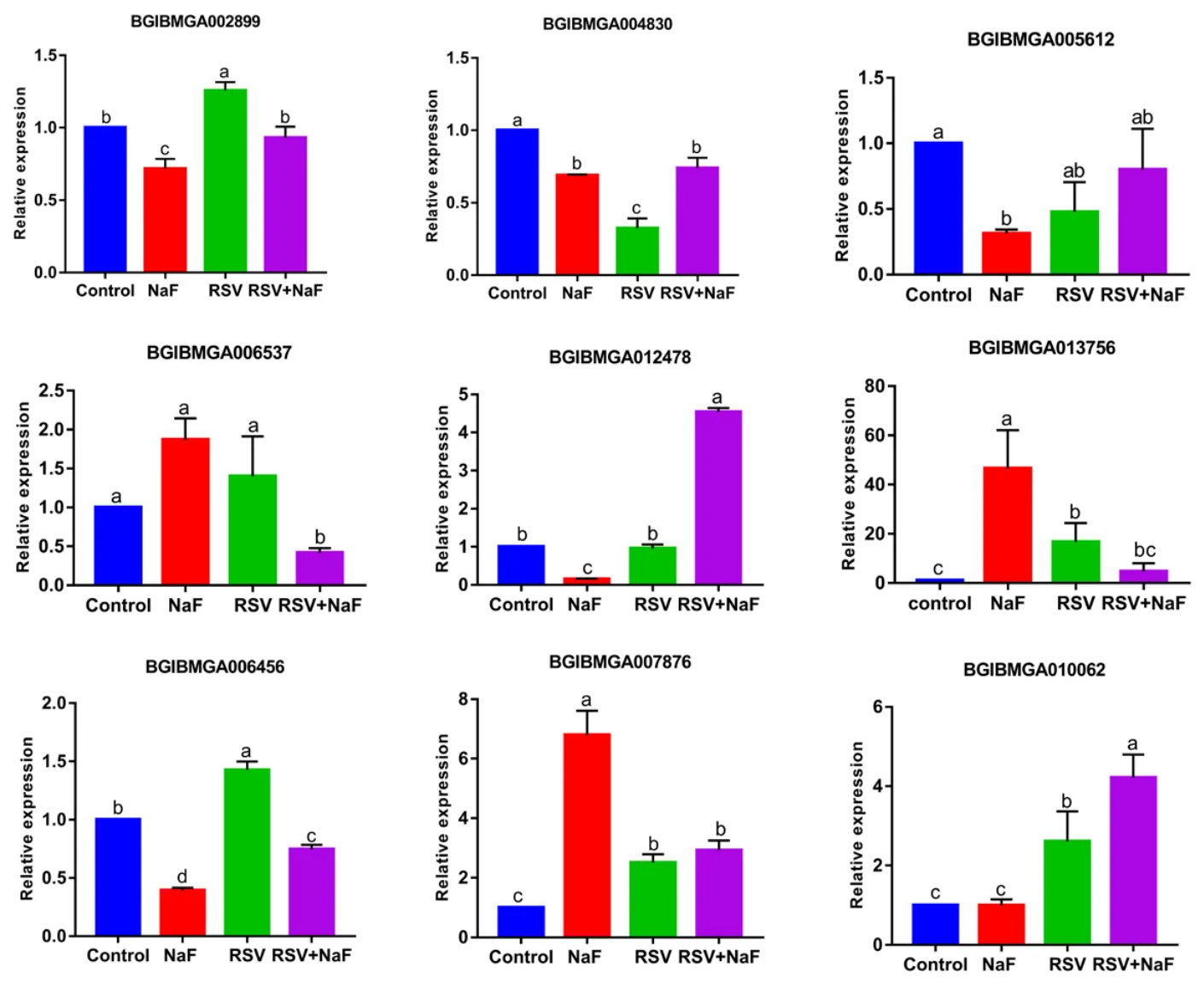
RT–qPCR validation of nine differentially expressed genes (DEGs). Group differences were assessed by one-way ANOVA followed by Fisher’s least significant difference (LSD) post hoc test. Statistical significance was set at *p* < 0.05. Bars without a common lowercase letter differ significantly (*p* < 0.05).

RT–qPCR validation. Nine DEGs were randomly selected for validation by RT–qPCR. Gene IDs and primer sequences are provided in Table S1, and BmGAPDH was used as the internal reference gene. As shown in Figure 3, the RT–qPCR results were consistent with the transcriptomic expression patterns, supporting the reliability of the RNA-seq dataset.

### 3.3. Screening, Functional Annotation, and Parallel Reaction Monitoring (PRM) Validation of DEPs

**Figure 4:**
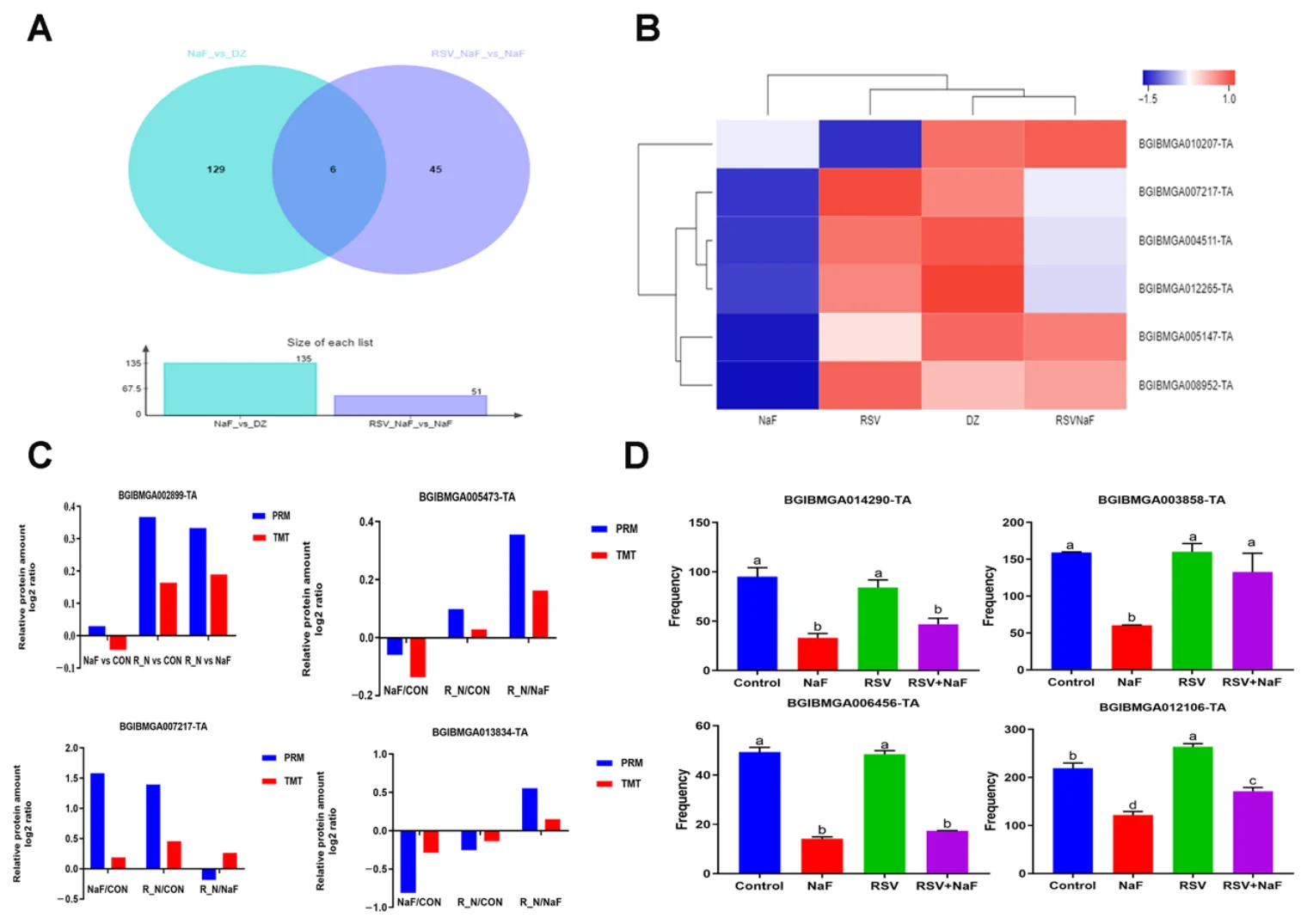
(**A**) Venn analysis of NaF_vs_DZ with RSV_NaF_vs_NaF protein set; (**B**) Cluster analysis of 6 DEPs; (**C**) PRM validation of DEPs; (**D**) Results of enzyme activity assay. ANOVA was used to compare the differences between the groups, LSD was used as a post hoc test, and *p* < 0.05 was generally regarded as a significant difference. Bars without a common lowercase letter differ significantly (*p* < 0.05).

Principal component analysis (PCA) of the TMT-based proteomic data (Figure S2) showed high reproducibility within groups and clear separation between the four experimental groups. Differentially expressed proteins (DEPs) were identified using the criteria of *p* < 0.05 and fold change (FC) > 1.1 or <0.83. For NaF_vs_DZ, 135 DEPs were detected, including 79 upregulated and 56 downregulated proteins. For RSV_NaF_vs_NaF, 51 DEPs were identified, of which 49 were upregulated and 2 were downregulated (Table S5). Venn analysis of NaF_vs_DZ and RSV_NaF_vs_NaF revealed six overlapping proteins (Figure 4A). Hierarchical clustering (Figure 4B) indicated that all six proteins were downregulated in the NaF group, whereas their expression was restored in the RSV+NaF group, suggesting that RSV may counteract NaF-induced suppression of these proteins.

#### 3.3.1. PRM Validation and Enzyme Activity Assay

In the PRM protein targeting validation experiment, we successfully validated the protein abundance quantification of four DEPs. Based on the relative expression of the corresponding peptide for each DEP across different sample groups, the relative expression differences in DEPs between groups were further calculated and compared using the TMT results, as depicted in Figure 4C,D. The PRM validation results largely aligned with the trends observed in the TMT results; however, there were still some discrepancies, indicating that different assay methods could introduce variability in the results of the assay.

Four enzymes (BGIBMGA014290-TA, BGIBMGA003858-TA, BGIBMGA006456-TA, and BGIBMGA012106-TA) were selected from the proteomes of NaF_vs_DZ and RSV_NaF_vs_NaF. The activities of all four enzymes were significantly lower in the NaF group than in the control group (*p* < 0.05; Figure 4D), and the addition of RSV partially restored their enzyme activities. The results of the ELISA enzyme activity assay generally correlated with the trend observed in the proteomics assay for the four sample groups. However, the magnitude of the activity changes did not fully parallel the TMT-derived protein-abundance changes, which may reflect regulation of catalytic activity by post-translational modifications, endogenous inhibitors, and substrate availability, as well as differences in the specificity and sensitivity of the two analytical methods.

A previous study conducted by Chen et al. jointly analyzed 1493 DEGs from the Control_vs_NaF gene set and 135 DEPs from the NaF_vs_DZ protein set, resulting in the identification of 13 genes and proteins with consistent expression. Similarly, the study also analyzed the 1091 DEGs in the NaF_vs_RSV_NaF gene set along with the 51 DEPs in the RSV_NaF_vs_NaF protein set, leading to the identification of 4 genes and proteins with consistent expression (Figure S3). Detailed information can be found in Table S6.

To further understand the biological functions of *BmeFE4*, we performed relevant biosignature analysis and RNA interference (RNAi) function prediction of *BmeFE4*.

#### 3.3.2. Results of the BmeFE4 Gene Sequence Analysis

Based on the transcriptomic data and the NCBI GenBank record for *BmeFE4* (accession no. XM_038013555.1), we determined that the silkworm *BmeFE4* transcript is 1750 nt in length and contains a 1602-nt open reading frame (ORF) encoding a 534-amino-acid protein. The 5′ and 3′ untranslated regions (UTRs) comprise 90 and 58 nt, respectively. The predicted molecular weight and isoelectric point (pI) of BmeFE4 are 59.5 kDa and 5.18. SignalP v5.0 predicted a N-terminal signal peptide (Figure S4A).

#### 3.3.3. Multiple Sequence Alignment and Phylogenetic Tree

The BmeFE4 amino acid sequence was aligned with carboxylesterases from other species, including the wild silkworm *Bombyx mandarina* (GenBank accession no. XP_028035522.1), *Maniola hyperantus* (accession no. XP_034838916.1), *Pieris rapae* (accession no. AQY62747.1), *Papilio xuthus* (accession no. KPI91074.1), and *Spodoptera frugiperda* (accession no. XP_035449350.1). As shown in Figure S4B, BmeFE4 shared 91.03%, 52.33%, 51.57%, 49.62%, and 50.58% sequence identity with the corresponding proteins from these species, respectively. To further elucidate the evolutionary relationships among insect carboxylesterases, a phylogenetic tree was constructed, resolving four major clades (Figure S4C). The clustering pattern indicated that BmeFE4 belongs to the α-esterase subgroup.

#### 3.3.4. Expression of *BmeFE4* in Different Developmental Periods and Different Tissues

**Figure 5:**
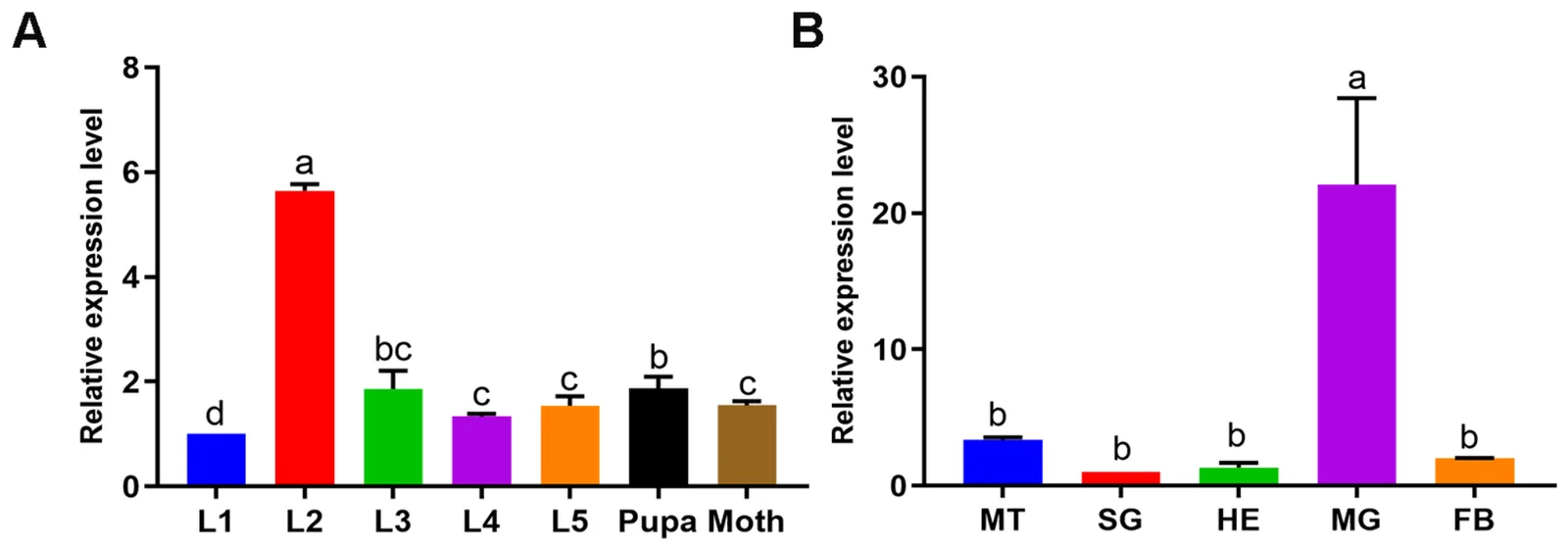
*BmeFE4* expression profiles determined by RT–qPCR. (**A**) Relative expression of *BmeFE4* across developmental stages. (**B**) Relative expression of *BmeFE4* in different tissues. MT, Malpighian tubule; SG, silk gland; HE, head; MG, midgut; FB, fat body. Data are shown as mean ± SD (*n* = 3). Statistical differences were evaluated by one-way ANOVA in SPSS v24.0, followed by Fisher’s least significant difference (LSD) post hoc test. *p* < 0.05 was considered statistically significant. Bars without a common lowercase letter differ significantly (*p* < 0.05).

RT–qPCR analysis showed that *BmeFE4* was expressed throughout development, although transcript abundance varied across stages. During the larval period, *BmeFE4* expression was lowest at the first instar (L1) and peaked at the second instar (L2), reaching approximately sixfold the L1 level. Expression subsequently decreased at L3, and no significant differences were observed among L3, L4, L5, and the adult (moth) stage (*p* ≥ 0.05). At the pupal stage, *BmeFE4* expression increased, but this increase was not statistically significant compared with L3 (*p* ≥ 0.05; Figure 5A). Tissue-specific expression was assessed in five tissues (marsupial duct, silk gland, head, midgut, and fat body). *BmeFE4* transcripts were most abundant in the midgut, followed by the marsupial duct, fat body, and head, with expression levels 22.10-, 3.39-, 2.01-, and 1.34-fold higher than those in the silk gland, respectively. Notably, midgut expression was significantly higher than that in the other tissues (*p* < 0.05), whereas no significant differences were detected among the Malpighian tubule, silk gland, head, and fat body (*p* ≥ 0.05; Figure 5B).

#### 3.3.5. siRNA-Mediated Knockdown Efficiency and Body-Weight Changes

**Figure 6:**
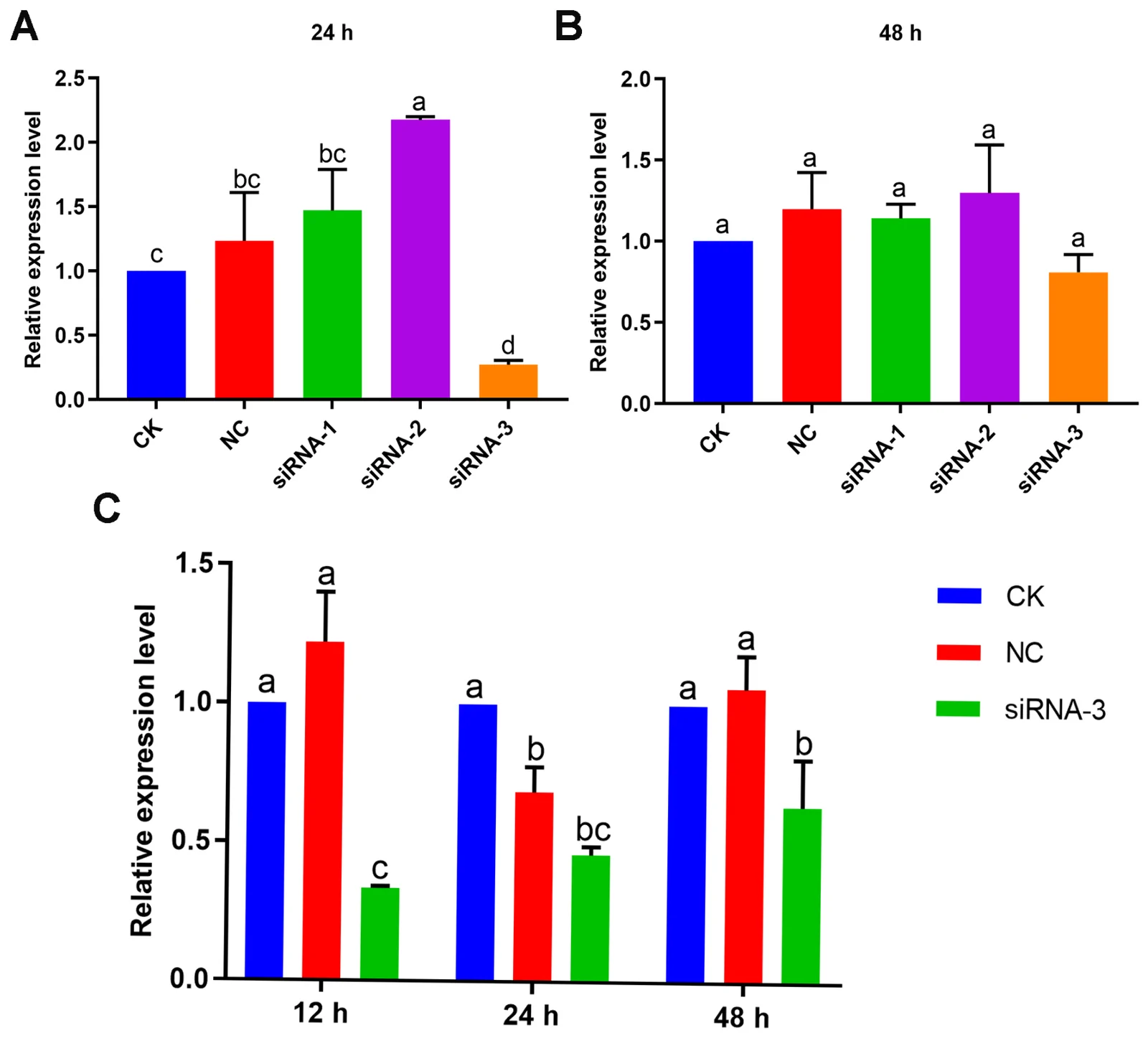
siRNA-mediated interference of *BmeFE4* expression. (**A**) *BmeFE4* transcript levels at 24 h after injection of siRNA-1, siRNA-2, or siRNA-3. (**B**) *BmeFE4* transcript levels at 48 h after injection of siRNA-1, siRNA-2, or siRNA-3. (**C**) Time-course of *BmeFE4* knockdown following siRNA-3 injection. Differences among groups were assessed by one-way ANOVA followed by Fisher’s least significant difference (LSD) post hoc test; *p* < 0.05 was considered statistically significant. Bars with no lowercase letter in common differ significantly (*p* < 0.05), whereas bars sharing at least one lowercase letter are not significantly different (*p* ≥ 0.05).

In a pilot experiment, three siRNA fragments (siRNA-1, siRNA-2, and siRNA-3) were designed and injected into day-1 fifth-instar larvae. Larvae were collected at 24 and 48 h post-injection to quantify *BmeFE4* transcript levels by RT–qPCR (Figure 6A,B). At 24 h, *BmeFE4* expression was higher, but not significantly, in the siRNA-1 group and significantly increased in the siRNA-2 group, whereas it was significantly reduced in the siRNA-3 group compared with the control (CK) group (*p* < 0.05; Figure 6A). A similar pattern was observed at 48 h: *BmeFE4* transcript abundance remained elevated in the siRNA-1 and siRNA-2 groups relative to CK, while it decreased in the siRNA-3 group; however, this reduction was not statistically significant at 48 h (Figure 6B). Collectively, these results indicate that siRNA-1 and siRNA-2 did not achieve effective knockdown, whereas siRNA-3 produced the most pronounced silencing of *BmeFE4* and was therefore selected for subsequent experiments.

To confirm the efficacy of siRNA-3, a second knockdown experiment was performed in fifth-instar larvae by injecting siRNA-3 and sampling individuals at 12, 24, and 48 h post-injection to quantify *BmeFE4* expression. RT-qPCR analysis (Figure 6C) showed that *BmeFE4* transcript levels were significantly reduced at all three time points compared with the CK group (*p* < 0.05). The strongest suppression was observed at 12 h, indicating that siRNA-3 achieved maximal knockdown shortly after injection. Accordingly, the non-significant reduction at 48 h in the preliminary screening may reflect experimental variability rather than an absence of siRNA-3 activity, as significant suppression at the same time point was confirmed in the independent validation experiment.

**Figure 7:**
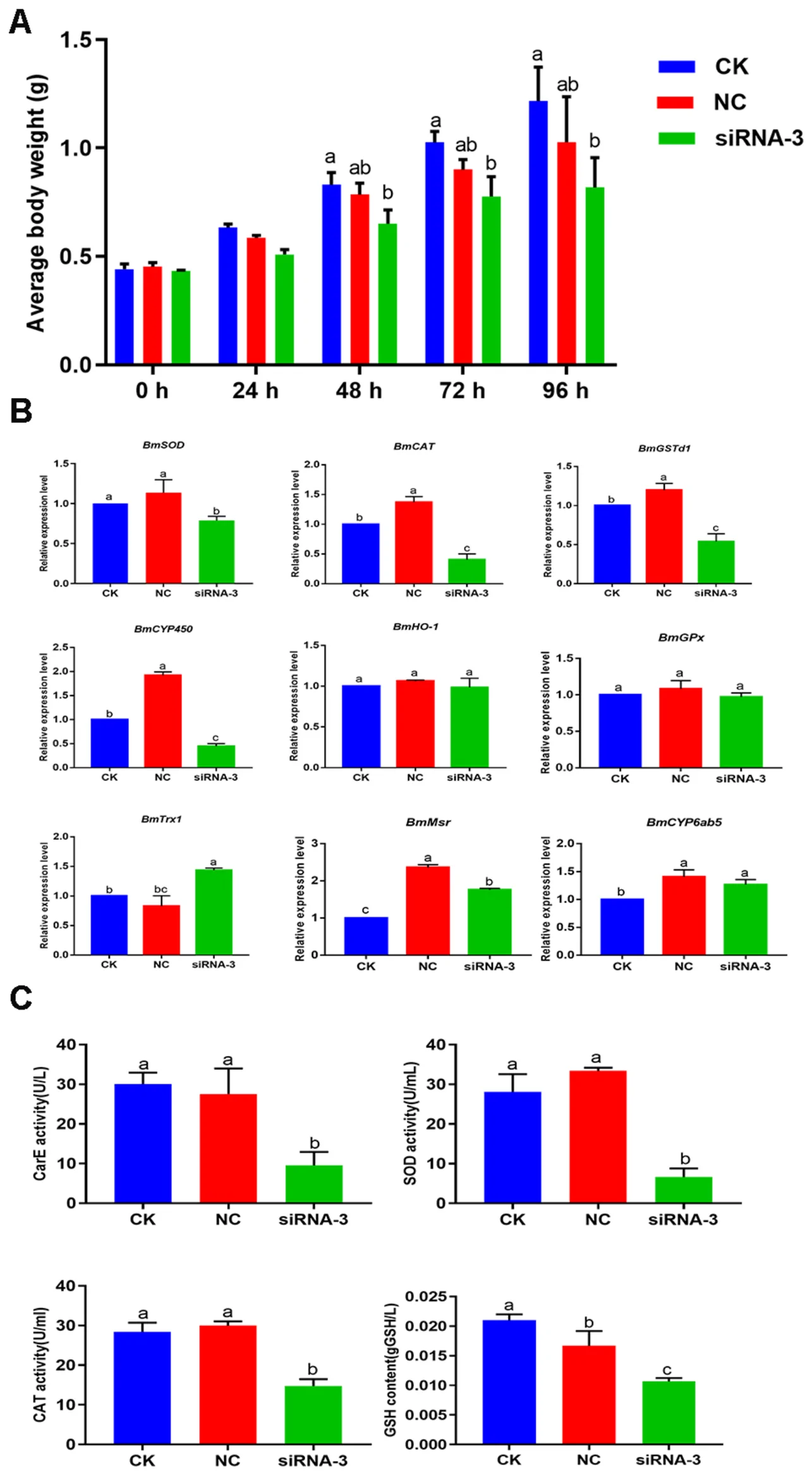
Effects of siRNA-3–mediated *BmeFE4* knockdown on larval growth and antioxidant status. (**A**) Changes in body weight of silkworm larvae following siRNA-3 injection. (**B**) Relative mRNA expression of antioxidant-related genes after siRNA-3 injection. (**C**) Carboxylesterase, SOD, and CAT activities and glutathione (GSH) content in larvae after siRNA-3 injection. Differences among groups were analyzed by one-way ANOVA followed by Fisher’s least significant difference (LSD) post hoc test; *p* < 0.05 was considered statistically significant. Bars without a common lowercase letter differ significantly (*p* < 0.05).

Fifth-instar larvae were sampled at 0, 12, 24, 48, 72, and 96 h after injection, and body weight was recorded at the same time each day to minimize diurnal variability. As shown in Figure 7A, during the first 24 h, larval weight in the NC and siRNA-3 groups showed a slight, non-significant decrease relative to the CK group (*p* ≥ 0.05). In contrast, at 48 and 72 h, larvae in the siRNA-3 group exhibited a significant reduction in body weight compared with CK (*p* < 0.05 and *p* < 0.01, respectively). By 96 h, body weight in the siRNA-3 group remained significantly lower than in the CK group (*p* < 0.001).

#### 3.3.6. Changes in Antioxidant-Related Gene Expression After *BmeFE4* Silencing

To further elucidate the role of *BmeFE4*, we examined whether *BmeFE4* knockdown affected the expression of antioxidant-related genes. RT–qPCR results are presented in Figure 7B. At 72 h after *BmeFE4* RNAi, the transcript levels of *BmSOD*, *BmCAT*, *BmGSTd1*, *BmCYP450*, *BmHO*-1, and *BmGPx* were decreased, whereas *BmTrx1*, *BmMsr*, and *BmCYP6ab5* were upregulated.

#### 3.3.7. Enzyme Activities in *BmeFE4* RNAi Larvae

To evaluate the functional consequences of *BmeFE4* knockdown, carboxylesterase activity was measured following siRNA-3 treatment (Figure 7C). Carboxylesterase activity was significantly reduced at 72 h post-interference (*p* < 0.05; Figure 7C). In addition, SOD and CAT activities, as well as glutathione (GSH) content, were lower in the siRNA-3 group than in the CK group.

## 4. Discussion

The research focus on fluoride toxicity among organisms has remained high in recent decades, involving studies on plants, animals, insects, and even humans [33,34,35]. Reactive oxygen species-mediated oxidative stress is a common feature of fluoride toxicity [36]. Fluoride also mediates signaling pathways, such as phosphoinositide 3-kinase, c-Jun N-terminal kinase, and mechanistic target of rapamycin, both ex vivo and in vivo, to induce autophagy [37,38] and apoptosis in certain tissue cells [39,40]. Fluorosis not only leads to systemic diseases [41] but also causes neurotoxic brain damage, which significantly impacts physical and mental health. Chronic fluorosis adversely affects learning, memory, and intelligence quotient in both animals and humans [8,42]. Moreover, overexposure to fluoride during childhood may permanently affect brain structure and function, leading to irreparable damage [43]. Consequently, researchers are continuously striving to identify methods and drugs that can alleviate or treat fluorosis. Li et al. discovered that sodium butyrate can ameliorate fluorosis-induced neurotoxicity in mice by influencing hippocampal glycolysis [44]. Exercise has been found to enhance anxiety and depression-like behavior in mice by increasing the synthesis of gamma-aminobutyric acid and transport-related proteins [45]. Natural and synthetic phytochemicals, such as riboflavin [46], lycopene [47], and selenium [48], have shown potential in alleviating fluoride-induced oxidative stress and tissue damage. Beyond small-molecule interventions, the endophytic bacterium *Bacillus altitudinis* WR10 alleviated Cu toxicity in wheat by enhancing reactive oxygen species scavenging and phenylpropanoid biosynthesis [49], whereas a polyanionic hyaluronic acid-based hydrogel reduced heavy-metal stress by removing Fe^3+^, Cd^2+^, and Pb^2+^ from contaminated soil and promoting sorghum growth [50], illustrating complementary biological and material-based strategies for mitigating environmental toxicant stress.

In this study, NaF exposure was found to cause tissue damage and oxidative stress disorders in organisms. RSV, a small molecule polyphenolic compound renowned for its antioxidative stress function [51,52], has been extensively studied for its potential mechanisms in senescence and prevention of age-related diseases [53,54]. However, due to its structural properties, RSV showed a low solubility in water (<0.05 mg/mL) [55]. Previous investigations using 14C-labeled RSV demonstrated that RSV is absorbed in the human body at approximately 70% [56]. Nonetheless, its limited bioavailability and pharmacokinetics hinder its practical applications [57,58]. These pharmacokinetic limitations may also restrict the broader clinical translation of RSV. Researchers have attempted to enhance its bioavailability based on drug delivery systems such as micelles and nanomaterials [59,60]. In the present study, we found that exposure to 5 mM RSV did not impede larval growth and development or induce severe damage to midgut tissues, and significantly ameliorated NaF-induced neurotoxicity caused by body weight loss, midgut tissue damage, and haphazard crawling in silkworms. Because RSV was administered before and during NaF exposure, these findings indicate a protective rather than a post-exposure therapeutic effect. Thus, we hypothesized that RSV may possess promising efficacy in alleviating fluoride toxicity in silkworms.

The genes Control_vs_NaF and NaF_vs_RSV_NaF were examined, focusing on differentially overlapping genes with opposite expression trends. The G1 gene set, involved in aminosugar and nucleotide sugar metabolism was significantly enriched, and it was found that aminosugar and nucleotide sugar metabolism were closely related to the hexosamine biosynthesis pathway (HBP) [61]. This pathway encodes BGIBMGA007517 glutamine-fructose-6-phosphate aminotransferase, which is a rate-limiting enzyme. Studies have confirmed that Glutamine-fructose-6-phosphate transaminase (GFAT) is associated with the procytokine transforming growth factor (TGF)-β1, suggesting that glucose metabolism influences TGF-β1 activity [62,63]. Therefore, we hypothesized that RSV inhibits the effects of NaF on cytokines by down-regulating GFAT expression, thereby attenuating damage to cell development. The gene BGIBMGA012188 encodes UDP-glucose-6-dehydrogenase, which is the rate-limiting enzyme of the pentose phosphate pathway. It regulates the production of reduced coenzyme II. The reduced form of nicotinamide adenine dinucleotide phosphate maintains GSH in its reduced form, thereby affecting the detoxification of ROS by GSH and interfering with the ability of the silkworm to resist oxidative stress [64,65]. The G2 gene, glucuronosyltransferase UGT (BGIBMGA003817, BGIBMGA003835, BGIBMGA010099, BGIBMGA010286, BGIBMGA010287, BGIBMGA010433, BGIBMGA013860), was annotated as an important phase II drug metabolism enzyme in the intersection set. It is involved in endogenous (e.g., fatty acids, bilirubin, etc.) and exogenous (e.g., drugs, pollutants) clearance, metabolism, and detoxification [66,67,68]. The enrichment results suggest that the interaction between NaF and RSV affects all of these genes and, to some extent, regulates the resistance to oxidative stress in the silkworm.

The TMT results revealed a low number of differential proteins obtained after threshold screening. Consequently, the protein-level analysis had poor correlation with the RNA-seq sequencing data. Although PRM and TMT showed broadly consistent expression trends, quantitative discrepancies may arise from differences in peptide selection, sample digestion, ionization efficiency, and data normalization between the two analytical platforms; therefore, these results should be interpreted cautiously. In the correlation analysis, the esterase FE4 (BGIBMGA002899), which was identified, plays an important role in both transcriptomics and proteomics in the regulation of NaF and RSV. Carboxylesterases serve as detoxifying enzymes that hydrolyze structurally diverse molecules [69]. They are involved in the biochemical mechanisms of pesticide resistance in certain pest species, reducing the uptake of organophosphates in the insect gut [70,71].

In this study, we observed that NaF affected the expression of carboxylesterase in *Bombyx mori* downregulating its expression and inhibiting its detoxification function, which made *Bombyx mori* susceptible to fluoride-induced fluorosis. On the other hand, ingestion of RSV significantly upregulates the expression of esterase FE4, suggesting that esterase FE4 may be an important target for the action of RSV. The antioxidant and detoxifying effects of esterase FE4 have been studied in a variety of aphids [72,73] as well as in insects such as the Asian citrus woodlouse and Asian citrus psyllid [74]. However, the specific mechanism of resistance has not yet been elucidated. In the present study, BmeFE4 demonstrated hydrolytic enzyme activity and catalytic activity. The validation of enzyme activity further confirmed that RSV could promote the expression of *BmeFE4*. Aa comprehensive comprehension understandingdditionally, RNAi-mediated knockdown of *BmeFE4* verified that reduced expression of *BmeFE4* decreased SOD and CAT activities and GSH content in the midgut tissues of *Bombyx mori*, thereby affecting its ability to resist oxidative stress. Several limitations of the present study should be acknowledged. The RNAi experiments neither employed a complete factorial design across the Control, NaF, RSV, and RSV+NaF conditions nor included a second independently effective siRNA. Thus, although the results support the involvement of *BmeFE4* in the observed response, they do not establish that *BmeFE4* is specifically required for RSV-mediated protection, and sequence-specific off-target effects cannot be excluded. In addition, 600 mg·L^−1^ denotes the nominal concentration of the NaF solution applied to mulberry leaves; because the fluoride retained by the treated leaves and individual food intake were not quantified, the actual fluoride dose ingested per larva remains unknown. Moreover, because vehicle-matched DMSO controls were not included, possible vehicle-related effects cannot be completely excluded. Future studies incorporating factorial RNAi designs, independent effective siRNAs, rescue experiments, vehicle-matched controls, and direct exposure measurements are warranted.

## 5. Conclusions

**Figure 8:**
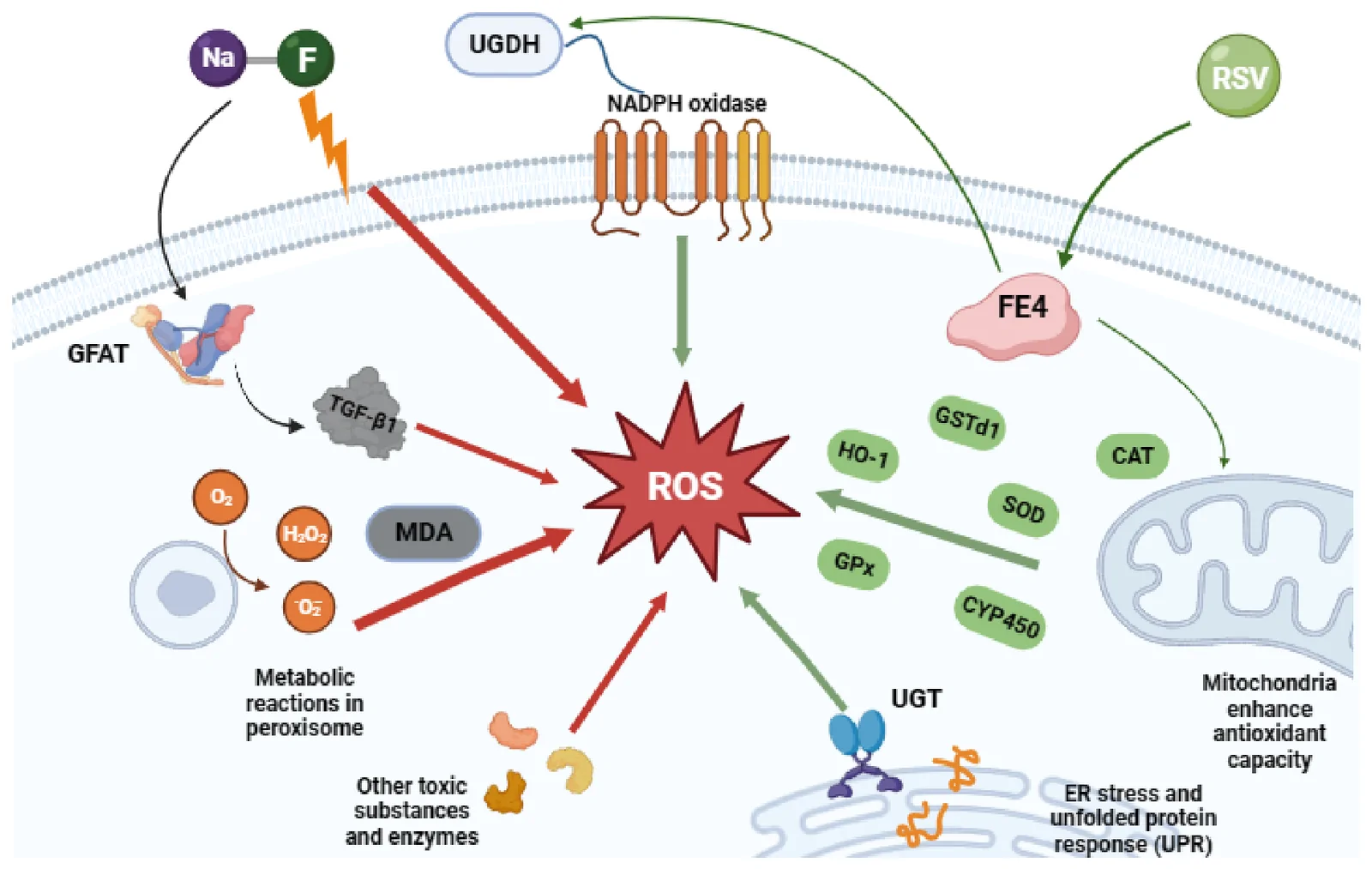
The potential mechanism of esterase BmeFE4 in reducing NaF-induced fluorosis in silkworms through Resveratrol. The figure was created in Biorender. Dali, D. (2026) https://BioRender.com/s8du29w. Arrows indicate the proposed direction of the relationships among the indicated molecules and pathways.

Integrating RNA-seq, TMT-based proteomics, and RNAi experiments, we demonstrate for the first time that resveratrol (RSV) mitigates NaF-induced fluorosis in silkworms. Specifically, RSV improved larval body weight and locomotor behavior, attenuated midgut histopathological injury, reversed the NaF-induced increase in malondialdehyde (MDA), restored SOD and CAT activities and glutathione (GSH) levels, and thereby alleviated oxidative stress (Figure 8). qPCR and PRM-based protein validation of the overlapping transcriptomic and proteomic datasets—showing concordant expression changes during intoxication and subsequent detoxification—identified BmeFE4 as a key detoxification-associated target. Consistently, *BmeFE4* knockdown reduced SOD and CAT activities and GSH content in *Bombyx mori* midgut tissue, indicating that BmeFE4 contributes to redox homeostasis. Collectively, these findings suggest that BmeFE4 mediates, at least in part, the toxic effects elicited by NaF.

Taken together, these results indicate that BmeFE4 plays a critical role in the silkworm response to fluoride-induced toxicity. Moreover, resveratrol (RSV) partially mitigated fluoride toxicity, at least in part, by modulating FE4 expression. Given its involvement in metabolic detoxification and oxidative stress regulation, FE4 represents a promising molecular target for the development of fluoride detoxification strategies.

## Data Availability

The original contributions presented in this study are included in the article/Supplementary Material. Further inquiries can be directed to the corresponding authors.

## Supplementary Materials

The following supporting information can be downloaded at: https://www.mdpi.com/article/10.3390/toxics14090829/s1, Figure S1: Venn analysis of differentially expressed genes in Control_vs_NaF and NaF_vs_RSV_NaF gene sets; Figure S2: Principal component analysis of TMT sequencing data from four groups of silkworm midgut tissues; Figure S3: Venn analysis of S2 NaF_vs_RSV_NaF vs. RSV_NaF_vs_NaF protein set; Figure S4: (A) Prediction result of signal peptide; (B) Amino acid sequence alignment results of *BmeFE4*. The black, red, and blue parts indicate consistent comparison results of 100%, 80%, and 50%, respectively; (C) Construction of phylogenetic tree of carboxylesterases from different species. The red box indicates the target sequence; Table S1: Gene ID and primer sequences for RT-qPCR; Table S2: siRNA sequence information; Table S3: Quality assessment and comparative analysis of RNA-seq data from midgut tissues of four groups of domestic silkworms, pre- and post- quality control; Table S4: Screening for significantly different proteins; Table S5: Screening of differentially expressed genes using NOIseq software; Table S6: Statistics and details of the 4 genes and proteins.
