# Supplementary material for: Role of Esterase BmeFE4 in Mediating the Alleviating Effects of Resveratrol on NaF-Induced Fluorosis in Bombyx mori
Source: Toxics. 2026 Sep 18;14(9):829. doi: 10.3390/toxics14090829 (PMC13611032; doi:10.3390/toxics14090829)
Supplement: Supplementary file 1 [file toxics-14-00829-s001.zip › toxics-4514117-supplementary.pdf]

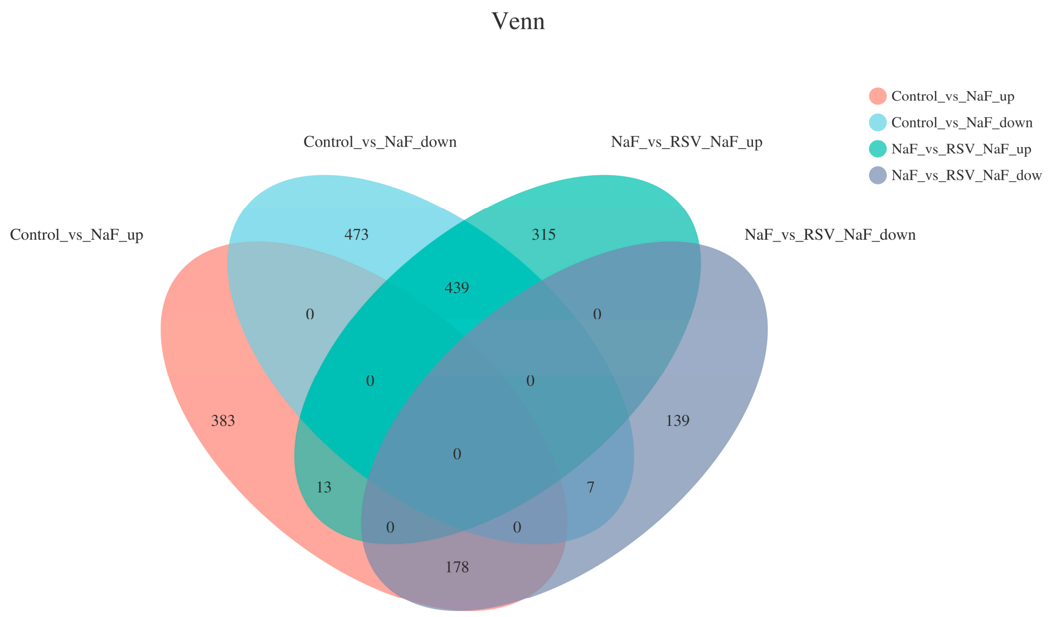

Figure S1. Venn analysis of differentially expressed genes in Control_vs_NaF and NaF_vs_RSV_NaF gene sets.

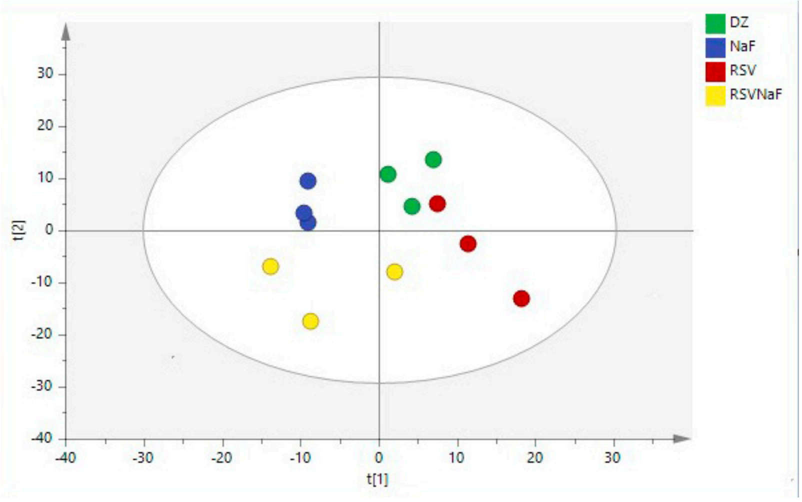

Figure S2. Principal component analysis of TMT sequencing data from four groups of silkworm midgut tissues.

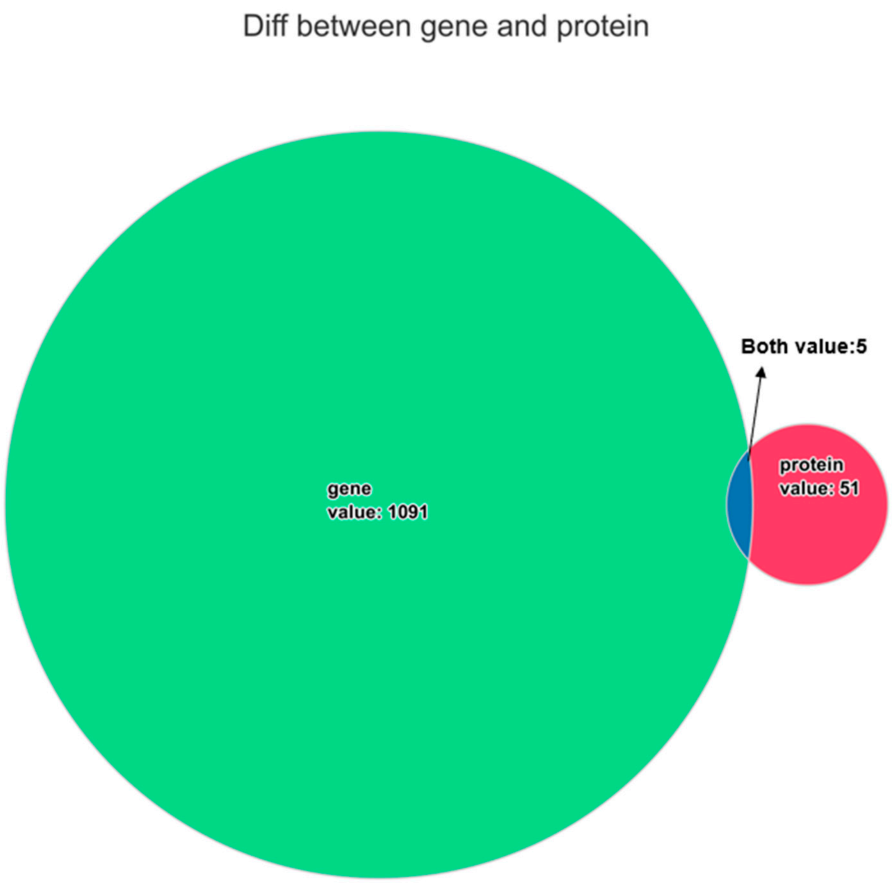

Figure S3. Venn analysis of S2 NaF_vs_RSV_NaF vs. RSV_NaF_vs_NaF protein set.

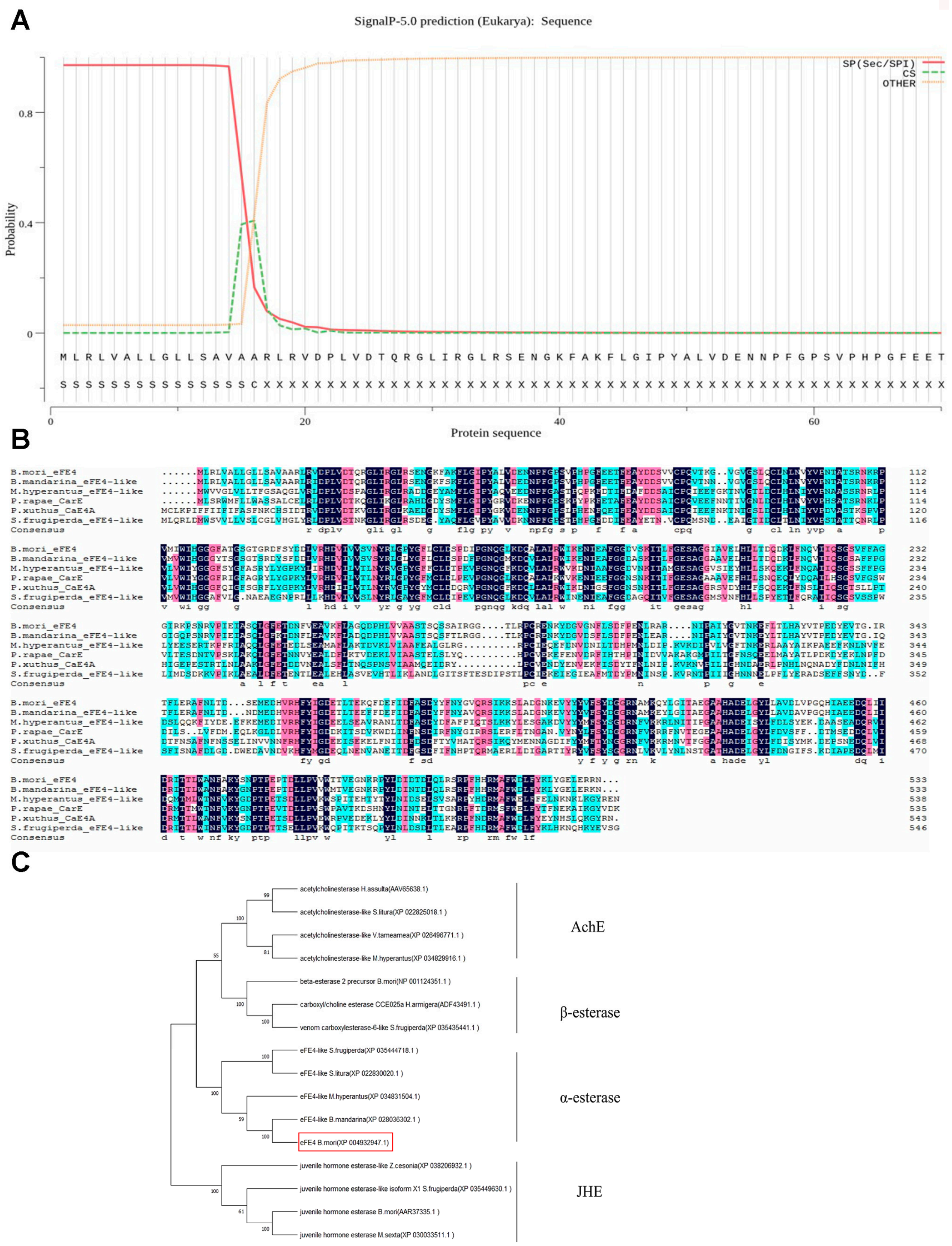

Figure S4. (A) Prediction result of signal peptide; (B) Amino acid sequence alignment results of *BmeFE4*. The black, red, and blue parts indicate consistent comparison results of 100%, 80%, and 50%, respectively; (C) Construction of phylogenetic tree of carboxylesterases from different species. The red box indicates the target sequence.

Table S1 Gene ID and primer sequences for RT-qPCR

Gene id	Forward primer	Reverse primer	Length(bp)

BGIBMGA002899	AGACGAGACGCTGACCGAGAAG	TAATAGACCGCTGGACGCCGTAG	89
BGIBMGA004830	TCACGCCTCCTGTCGCTCTATAC	CACATGCGGTCCTCGTCAATGG	145
BGIBMGA005612	GCCGTCAACAATCTGAATCAACCTG	TCTCCTTGATCTGTCGCCTCCTG	88
BGIBMGA006537	ACCGCCTTGTCGTGCTGTTATG	CCATAGTCGGGATTGTGTGCTGAG	140
BGIBMGA012478	CTCGGGCGTATTTGCGGATTACC	CCAGGACACGACACCGATTAGAAC	140
BGIBMGA013756	ACACTCTTCACCACGAATCTATTCAGG	TGGAGCAAGGGAAATTGTTGAGGTC	123
BGIBMGA006456	GGTGGAATGGCGGATAGACTAACAG	TGGCAAGGGTATCGGCAATTTGG	84
BGIBMGA007876	GAGAGGGCAGCAGAGCATTGTC	TGGCAGTCTTGAATATCGGCGTTC	147
BGIBMGA010062	ACGAGTAACACATGCCATCCACTTC	AGATGCACTGCCACCTTGAACG	136
BmGAPDH	AGGTCAAGGAGGCAGCGAAGG	GAAATTCCAGCGGCAGCATCAAAG	124

Table S2 siRNA sequence information

**Primers**	**Sense（5'→3'）**	**Antisense（5'→3'）**
siRNA-1	GGACGACAGUAGAAGGUAATT	UUACCUUCUACUGUCGUCCTT
siRNA-2	GCUUGGACAGUCCUGAUAUTT	AUAUCAGGACUGUCCAAGCTT
siRNA-3	GCAGUUCGAUGAGUUUAUATT	UAUAAACUCAUCGAACUGCTT
NC	UUCUCCGAACGUGUCACGUTT	ACGUGACACGUUCGGAGAATT

Table S3 Quality assessment and comparative analysis of RNA-seq data from midgut tissues of four groups of domestic silkworms, pre- and post- quality control

**Sample**	**Clean reads**	**Clean bases**	**Error rate (%)**	**Q20 (%)**	**Q30 (%)**	**Total mapped**	**Multiple mapped**	**Uniquely mapped**
Control[1,2]	47034267	7018919228	0.0238	98.50	95.28	43049437	5796936	37252501
NaF[1]	48162447	7163146148	0.0234	98.68	95.77	44467179	5497627.667	38766841
RSV[2]	49570958	7382679316	0.0235	98.63	95.63	45888222	6745189	39143033
RSV_NaF	47210008	7040395924	0.0240	98.46	95.16	43382321	6433424.333	36948897

Table S4 Screening for significantly different proteins

	**Total**	**Up**	**Down**
NaF_vs_DZ	135	79	56
RSV_NaF_vs_NaF	51	49	2

Table S5 Screening of differentially expressed genes using NOIseq software

	**Total**	**Up**	**Down**
Control_vs_NaF	1493	574	919
NaF_vs_RSV_NaF	1091	767	324

Table S6 Statistics and details of the 4 genes and proteins

**Expression pattern**	**Gene id**	**Description**	**Gene**	**Protein**
			Fold change	Fold change
Up-regulation	*BGIBMGA009194*	organic cation transporter protein	2.480	1.260651
	BGIBMGA004830	carboxypeptidase B	2.719	1.361477
	BGIBMGA005938	uncharacterized protein LOC101739791 isoform X1	2.577	1.170254
	BGIBMGA002899	esterase FE4-like	4.780	1.173096
